# Effects of antimicrobial nanocomposite films packaging on the postharvest quality and spoilage bacterial communities of mushrooms (*Chanterelles*)

**DOI:** 10.1016/j.fochx.2023.100996

**Published:** 2023-11-10

**Authors:** Kai Jiang, Bifen Zhu, Yudi Liu, Haiyan Chen, Mingwei Yuan, Yuyue Qin, Margaret Brennan, Charles Brennan

**Affiliations:** a Faculty of Food Science and Engineering, Kunming University of Science and Technology, Kunming 650550, China; b Green Preparation Technology of Biobased Materials National & Local Joint Engineering Research Center, Yunnan Minzu University, Kunming 650500, China; c School of Science, Royal Melbourne Institute of Technology University, Melbourne 3000, Australia

**Keywords:** Mesoporous nanosilica, Citral essential oil, *Chanterelles*, Preservation, Bacterial flora changes

## Abstract

Poly (lactic acid) (PLA) composite films with the addition of mesoporous silica nanoparticles MSN (0, 2, 4 and 6 wt%) loaded with 10 wt% citral (CIT) were prepared for application in *Chanterelles* packaging. Composite films with added MSN/CIT showed good mechanical properties, especially 4MSN/CIT/PLA. Changes in physicochemical properties and bacterial flora of *Chanterelles* during packaging and storage were tested. Compared with CIT/PLA, *Chanterelles* packed with 4MSN/CIT/PLA showed about 1.62-times lower browning value, 1.53-times lower electrolyte permeability, and 1.83- and 1.78-times lower PPO and POD, respectively, at 12 day. Better physicochemical properties of *Chanterelles* can be maintained. For bacterial flora changes, *Chanterelles* packaged with 4MSN/CIT/PLA had more stable flora (*p* < 0.05) and lower species diversity during storage (*p* < 0.05), effectively controlling the growth and reproduction of their dominant spoilage bacteria (*Enterobacteriaceae spp*). In conclusion, the composite membranes obtained by the addition of MSN/CIT to PLA have great potential in the storage of *Chanterelles.*

## Highlights

• Citral was loaded into the film as a bioactive antimicrobial agent.
• The MSN was used as a carrier of antimicrobial agent.
• Composite film can better maintain the physiological activity of *Chanterells*.
• Composite film enhances PPO and POD activities of *Chanterelles*.
• Composite film had a significant effect on the microbial changes of *Chanterelles*.

## 1 Introduction

*Chanterelles*, belongs to the *Cantharellaceae* family and is harvested during summer and autumn. They are natural food source with excellent taste and rich in nutrients and have a long history of edible and medicinal use. Research shown that *Chanterelles* have antioxidant, antihypertensive, immunomodulatory, anti-inflammatory, antiviral, anti-hypoxic and inhibitory effect on the angiotensin-converting enzyme (Nowakowski et al., 2021). Studies show that *Chanerelles* contain protein, fat, carbohydrates, and mineral salts such as selenium and vitamins, show health-promoting effects (Zawadzka et al., 2022). However, *Chanterelles* is highly susceptible to moisture loss and microbial contamination during storage. Therefore, developing efficient methods to inhibit the microbial contamination and maintain quality of *Chanterelles* during storage is urgently needed and is of great importance to both the industry and consumers.

Essential oils (EOs) are safe, non-toxic active compounds derived from plants that have some antimicrobial activity (Cui et al., 2021). The Food and Agriculture Organization of the United Nations (FAO/WHO) have given Eos the designation of “Generally Recognized as Safe” (GRAS). (Liu et al., 2023). Citral is the main active components of the essential oils and have significant inhibitory effects on Gram-positive and Gram-negative bacteria and fungi (Tao et al., 2023). However, citral are volatile, unstable, and have a strong aroma.

Mesoporous silica with high porosity, parallel-oriented nanochannels, homogeneous pores, and isolated material. It is therefore capable of loading a large number of antimicrobial active substances. Yan et al., (2023) discovered that packaging mushrooms with films containing oregano essential oil (OEO) and mesoporous nanosilica (MNS) significantly decreased weight loss, respiration rate, relative leakage rate, and polyphenol oxidase, as well as the aging of the mushrooms (*Agaricus bisporus*). Si et al., (2021) combined mesoporous silica nanoparticles (MSN) with capsaicin in meat preservation. The results showed that the release of capsaicin was slow, which resulted in stronger antioxidant properties due to the controlled release of capsaicin from the MSN with small particle size. Sattary et al., (2020) mixed lemongrass and clove oils with MSN for effective control of total blight in wheat crops. These studies have demonstrated that MSN has potential applications in terms of loading and slow-release effects on active antimicrobial substances. Thus, in the field of food packaging, food products can achieve extended shelf life and prevent oxidation.

To best of our knowneledge, there is little research about MSN load citral combined with PLA to develop an antibacterial composite film and used to preserve *Chanterelles.*

In this study, citral was loaded into the mesopores of MSN to ensure the stability of citral. A composite film based on PLA and MSN loaded citral was developed. The effects of composite packaging on the quality characteristics of mushrooms were examined by analyzing the weight loss, hardness, electrolyte permeability, polyphenol oxidase, catalase, and microbiology of *Chanterelles* during storage. In addition, high-throughput sequencing technology was utilized to analyze the changes of microbial colonies on the surface of *Chanterelles* during storage. This study evaluated the effect of active films on the post-harvest quality of *Chanterelles* and provided a theoretical basis for their application in food packaging.

## 2 Materials and methods

### 2.1 Materials and chemical reagents

Fresh *Chanterelles* were purchased from Shuimuhua Market (Kunming, China). Purchased *Chanterelles* were stored in a 4 °C condition and delivered right away to the lab. Mushrooms with uniform shape and size (stalk length 4–6 cm, thickness 0.4–0.5 mm, high maturity (uniform and dark yellow color)) and no mechanical damage were selected for the experiment. Citral (3,7-dimethyl-2,6-octadienal) was obtained from Senghe Chemical Reagent Co., ltd (Jinan, China) and of food grade. Tetraethyl orthosilicate, cetyl trimethyl ammonium bromide (CTAB), ammonium hydroxide (NH_3_·H_2_O), and acetyltributyl citrate (ATBC) were purchased from Aladdin Biochemicals & Technology Co Ltd (Shanghai, China). Dichloromethane was obtained from Chengdu kelong chemical Co., ltd (Sichuan, China). All other reagents were analytical grade.

### 2.2 Preparation of the nano-composite films

Mesoporous silica (MSN) was prepared according Lu et al., (2021). In brief, deionized water (100 mL) was mixed with CTAB (4.8 g), analytical-grade ethanol (100 mL), and aqueous ammonia (24 mL). TEOS (6.8 g) was added dropwise after the solution had been swirled for 10 min, and the mixture was then left to stir for a further 2 h at 25 °C. The solution was filtered to remove the solid particles, which were then cleaned with deionized water and dried at 100 °C. The sample received calcination treatment to remove the template. The nano-composite films were prepared as follows (Lu et al., 2021). Briefly, MSN (0, 2, 4 and 6 wt% based on PLA polymer matrix) and citral (10 wt%) were sonicated in 15 mL of dichloromethane solvent for 30 min, and the sonication was repeated three times by continuing to add 30 mL after the dichloromethane was dry. Then, 2 g PLA and 10 wt% acetyl tributyl citrate plasticizer (ATBC) were added in 50 mL dichloromethane to dissolve and mix well, and sonicated and exhausted to obtain the film-forming solution. Each 50 mL of film-forming solution was poured onto a (20 cm × 20 cm) PTFE plate and dried at 30 °C to obtain the composite film. And named as CIT/PLA film, 2MSN/CIT/PLA film, 4MSN/CIT/PLA film, and 6MSN/CIT/PLA film, respectively.

### 2.3 Performance testing of composite films

#### 2.3.1 Mechanical properties

The mechanical properties (tensile strength TS, modulus of elasticity EM and elongation at break ε) of the antimicrobial composite films were tested according to ASTM standard method D638-22 (ASTM, 2022) standard method and were measured by an electronic universal testing machine (model CMT4104, MTS Systems Co., Ltd, China). The samples were cut into rectangles of 150 mm × 10 mm, the initial grip spacing, and crosshead speed were set at 100 mm and 50 mm/min, respectively. And the thickness of the samples was measured using a spiral micrometer, three points of each sample were measured and averaged.

#### 2.3.2 Water vapor permeability (WVP)

The WVP was determined according to ASTM standard method E96-95 (Cui et al., 2021). First, the samples were placed at 25 °C and 50 % relative humidity for 48 h before the test. A certain amount of anhydrous silica gel (25 g) was weighed into a weighing bottle, and the film samples were sealed the weighing bottle using adhesive tape. The sealed weighing bottle was placed in a desiccator with saturated sodium chloride solution, and the desiccator was moved into a constant temperature and humidity chamber, and the temperature was set at 25 °C and the relative humidity at 50 %. The weight of the weighing bottle was measured every 1 h for 12 h. After that, the weight was measured every 12 h until the weight difference was stable. The WVP of the sample was calculated according to the following equation (Zhang et al., 2019).

(1) $$\begin{array}{c} \mathrm{wvp}=\frac{\Delta m\times t}{\Delta T\times A\times \Delta P} \end{array}$$

Δm represents the weight difference of the weighing bottle (g), t represents the average thickness of the sample (mm), ΔT represents the time interval (h) when the weight difference is stable, *A* represents the area of the weighing bottle opening (m^2^), and ΔP represents the difference in water vapor pressure (KPa) between the two sides of the film.

#### 2.3.3 Oxygen transmission rate (OTR)

Slightly modified from the original method (Guzman-Puyol et al., 2022). The OTR value of the composite membrane was measured using the oxygen analyzer system-Oxysense 5250I. The temperature was set to 23 °C and the humidity to 50 %. The oxygen pressure at the inlet is 5 bar. The sensing wells are blown with nitrogen and the drive wells are linked in air. The samples were cut to a size of 80 mm × 80 mm for testing. Each sample was tested three times and the average value was taken.

### 2.4 Mushroom packaging

The mushroom samples were packed by films and divided into four groups as follow: CIT/PLA group, 2MSN/CIT/PLA group, 4MSN/CIT/PLA group, and 6MSN/CIT/PLA group. The mushroom samples were stored at 4 ± 1 °C and evaluated for physiological indices at 0, 3, 6, 9 and 12 days, as well as changes in bacterial flora of samples packed in CIT/PLA and 4MSN/CIT/PLA groups were explored.

#### 2.4.1 wt loss

The weight loss of mushrooms of each packet was measured before and after storage of the experiment. For each treatment group the weight loss is calculated as follows:

(2) $$\begin{array}{c} \mathrm{Weightloss}=\frac{m_{1}-m_{2}}{m_{1}}\times 100 \end{array}$$

where *m_1_* was the weight on the first day, *m_2_* was the mass on each sampling day.

#### 2.4.2 Hardness

The hardness of mushrooms was evaluated on the mushroom cap using a texture analyzer (TA-XT Plus, LOTUN SCIENCE Co., SMS, Shanghai, China) and a P/36R probe was used for the hardness test. The speed of the probe was 2 mm/s and depth was 5 mm (Liu et al., 2019). The measurements were taken for five mushrooms per treatment.

#### 2.4.3 Color

The color of mushrooms cap was measured by a CR-400 Chroma Meter. The samples were selected randomly from each treatment group every 3 days to assess color change. L*, *a* *, and b* of samples were measured. The following equation was used to compute the browning index (BI) (Zhu et al., 2022):

(3) $$\begin{array}{c} x=\frac{a^{*}+1.75L^{*}}{5.645L^{*}+a^{*}-3.012b^{*}} \end{array}$$

(4) $$\begin{array}{c} \mathrm{BI}=\frac{\left[100\times \left(x-31\right)\right]}{0.172} \end{array}$$

#### 2.4.4 Electrolyte leakage

According to the methodology of Xu et al. (2021), The electrolyte leakage of mushrooms was measured by a DDS-11A Conductivity Meter. The mushroom was sliced, and added 20 mL of distilled water, shake vigorously, and measured the conductivity, noted *P*_1_*.* After boiling it for 10 min, cooling the sample to room temperature and, the conductivity was tested and noted *P*_2_., and the electrolyte permeability was determined using the following formula:

(5) $$\begin{array}{c} \mathrm{Electrolyte}\;leakage\left(\%\right)=\frac{P_{1}}{P_{2}}\times 100\% \end{array}$$

#### 2.4.5 PPO and POD

Measurement of PPO and POD in mushrooms was improved according to the method of Gong et al., (2023). The samples (3.0 g) were homogenized in 6 mL 0.05 mol phosphate buffer (pH 6.8). Solution was centrifuged at 8000*g*, 4 °C for 20 min, then the supernatant was collected. To measure PPO and POD activity, 0.2 mL enzyme extract was added to 4.4 mL of phosphate buffer substrate (100 mM sodium phosphate, pH 6.8, 0.4 mL guaiacol, 0.2 mL H_2_O_2_). The change in absorbance at 420 nm was measured.

#### 2.4.6 DNA extraction

Total bacterial DNA of *Chanterelles* packed in 4MSN/CIT/PLA and CIT/PLA composite films were extracted at 0, 3, 6, 9 and 12 days of storage, respectively. According to the original method with slight modification (Jiang et al., 2023). 5 g of the sample was cut into small pieces and mixed in a sterile beaker containing 25mLPBS buffer solution, and shaken in a shaker at 100rmp, 25 °C for 30 min. After standing for 10 min, the supernatant was centrifuged at 7000g for 30 min to obtain the precipitate containing microorganisms. As directed by the total bacterial DNA extraction kit (Sangon Biotech Co., Ltd., China), extract the mushrooms' complete bacterial DNA. UV–Vis spectrophotometer at a wave-length of 600 nm was used to measure the final concentration and purity of the DNA, and 1 % agarose gel electrophoresis was used to verify the DNA's quality. The V3- V4 high variant portion of the bacterial 16S rRNA gene was amplified using primers.341F: (5′-CCTAYGGGRBGCASCAG-3′) and 806 R (5′-GGACTACNNGGGTATCTAAT-3′). The PCR products were sequenced by Sangon Biotech Co., Ltd., China. The GenBank Sequence database was ultimately used to examine the sequencing data in order to ascertain the microbial composition.

By categorizing the valid series in the RDP database (https://rdp.cme.msu.edu/misc/resources.jsp) and clustering them into operational taxonomic units (OTUs), where the similarity ≥97 %. The Mothur program (version 3.8.31) was used to calculate the Chao1, Simpson, and Shannon indices.

### 2.5 Statistical analysis

To ensure data accuracy, data from each sample were replicated three times and the mean was calculated and the results were expressed as mean ± standard deviation. Data were analyzed by SPSS version 10.0 software. The *p*-Duncan multiple comparison method was used to estimate significant differences between means (*p* < 0.05). Data were visualized by using Origin 9.0. Principal coordinate analysis (PCoA) was visualized using https://www.chiplot.online/.

## 3 Results and discussion

### 3.1 Mechanical properties and WVP of composite films

The mechanical properties were shown in Table 1. the film thickness ranges from 63.2 to 68.3 μm, TS ranges from 11.8 to 19.4 MPa, EM ranges from 675 to 1198 MPa and ε ranges from 26.4 to 31.2 %.

**Table 1:** Thickness, mechanical properties WVP and OTR of composite films.

Films	CIT/PLA	2MSN/CIT/PLA	4MSN/CIT/PLA	6MSN/CIT/PLA
Thickness (μm)	63.2 ± 4.92^a^	67.6 ± 4.24^a^	66.8 ± 3.57^a^	68.3 ± 6.53^ab^
TS (MPa)	19.4 ± 0.74^d^	16.3 ± 0.21^c^	13.6 ± 0.14^b^	11.8 ± 0.16^a^
ε (%)	26.4 ± 0.57^a^	28.6 ± 0.28^a^	31.2 ± 0.21^b^	30.7 ± 1.12^b^
EM (MPa)	876 ± 4.11^b^	1198 ± 3.62^c^	812 ± 0.63^b^	676 ± 3.25^a^
WVP × 10^−2^(g·mm/m^2^·h·KPa)	0.88 ± 0.01^a^	0.95 ± 0.01^a^	1.56 ± 0.03^b^	2.41 ± 0.97^c^
OTR [(cm^3^/(24 h × m^2^)]×(cm/bar)	2.13 ± 0.07^c^	2.34 ± 0.06^b^	2.47 ± 0.02^a^	2.68 ± 0.12^a^

Data are presented as mean ± standard deviation and different letters (a - d) within the columns shows the significant differences (*p* < 0.05), where a is the lowest value.

The TS and EM of the 2MSN/CIT/PLA, 4MSN/CIT/PLA, and 6MSN/CIT/PLA composite films exhibited a lowering trend and ε showed an increasing trend (*p* < 0.05) when compared to the CIT/PLA film. The composite film made of 6MSN/CIT/PLA has the greatest and the lowest TS and EM. The composite films made of 6MSN/CIT/PLA showed the largest and smallest TS and EM, respectively. The TS and EM of 6MSN/CIT/PLA composite films decreased (*p* < 0.05) by 39.2 % and 22.9 %, respectively, while ε increased (*p* < 0.05) by 14.0 % compared to CIT/PLA films. The addition of MSN/CIT helps to improve the mechanical properties, but excessive MSN/CIT may lead to uneven dispersion and decrease mechanical properties of composite films. This might be because CIT, which was liquid at room temperature and was present in the film as oil droplets, reduced PLA intermolecular interactions and polymer chain interactions, partially substituting the stronger polymer–polymer interactions with weaker polymer-oil interactions, weakening the PLA network structure, and increasing the ductility of the film (Sharma et al., 2022). As a result, the TS and EM of the films were significantly reduced (*p* < 0.05) and ε increased (*p* < 0.05). Sharma et al., (2022) prepared thyme essential oil poly (3-hydroxybutyrate-*co*-4-hydroxybutyrate) composite films, and they believed the change in mechanical properties of the composite films due to the addition of essential oil to the incorporation of thyme oil located mainly between the polymer chains of P (3HB-*co*-4HB), weakening the intermolecular chain which maintains the adjacent interaction forces. The movement of the polymer chains was promoted, which increased the ε and the TS of the films.

The WVP values of composite films were shown in Table1. It can be seen that the WVP of composite films range from 0.88 to 2.41 × 10^-2^ g mm/m^2^ h KPa. Compared with the CIT/PLA film, the WVP values of 2MSN/CIT/PLA, 4MSN/CIT/PLA, and 6MSN/CIT/PLA composite films increased (*p* < 0.05) by 7.95 %, 77.3 %, and 173.9 %, respectively. For the 2MSN/CIT/PLA, 4MSN/CIT/PLA, and 6MSN/CIT/PLA composite films, the WVP increased with the increase of MSN/CIT concentration. The 6MSN/CIT/PLA film had the highest WVP value. This may be due to the fact that oils, when used as plasticizers, changed the three-dimensional molecular organization matrix of the film, increased free volume and chain flow, improved the interfacial interaction matrix between polymers, and decreased intermolecular gravitational forces (Dong et al., 2018). And as the molecular weight increased, it lead to an increase in porosity, which allowed more water to pass through the film, thus increased the WVP (Lu et al., 2022). In addition, oils prevented water molecules from interacting with polymer chains, increasing WVP (Qin et al., 2015).

As far as the OTR value is concerned, the OTR value increased (*p* < 0.05) with the increase in MSN. This is consistent with the fact that an increase in molecular weight leads to an increase in film porosity, which increases the OTR value, which is also consistent with leading to a change in the WVP value. Lu et al., (2022) showed a similar finding that the OTR values of low-density polyethylene (LDPE) films increased (*p* < 0.05) significantly under the influence of mesopores of halloysite nanotubes.

### 3.2 Changes in Chanterelles during storage

#### 3.2.1 Morphological changes

**Fig. 1:**
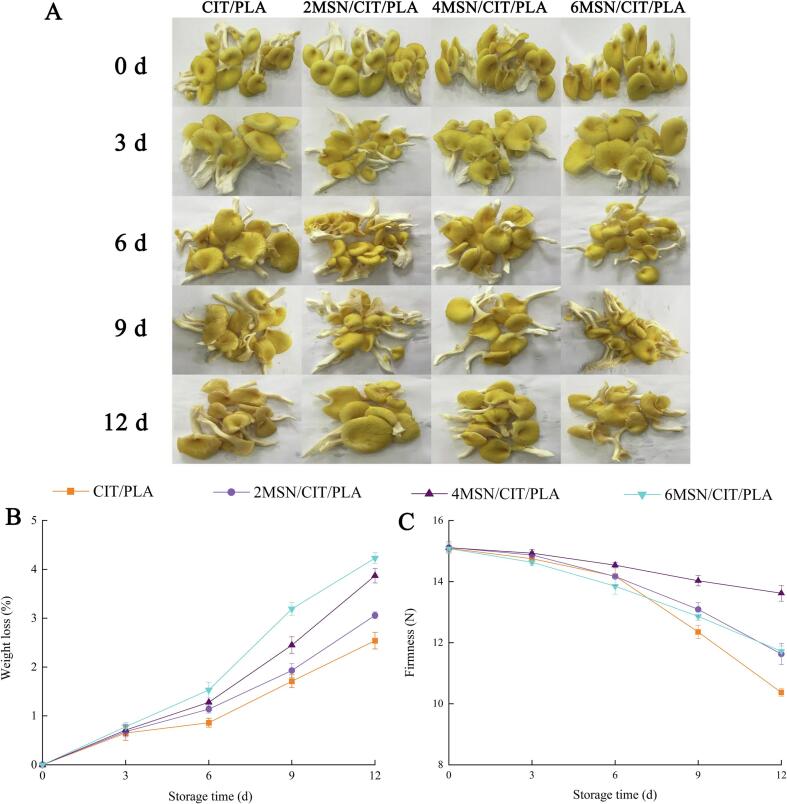
The changes in morphological (A), weight loss (B), and firmness (C) of *Chanterelles* during storage.

Morphological changes of Chanterelles during storage are showed in Fig. 1A. Fresh Chanterelles were bright in color and full in texture; while the color darkened and became dry with the increase of storage days, especially from the 6 day onwards. The morphology of Chanterelles in 4MSN/CIT/PLA, and 6MSN/CIT/PLA was better. We can judge this by the physiological indicators studied below.

#### 3.2.2 Weight loss

Transpiration and respiration cause fruits and vegetables to lose weight while in storage. Chanterelles treated with 2MSN/CIT/PLA, 4MSN/CIT/PLA, 6MSN/CIT/PLA, and CIT/PLA composite films all had an increase in weight loss during the course of storage (Fig. 1B).

The weight loss of *Chanterelles* was mainly due to the higher transpiration rate and the thin epidermal structure which could not prevent its rapid dehydration (Zhang et al., 2019). Weight loss was significantly higher (*p* < 0.05) in several groups of MSN/CIT/PLA compared to the CIT/PLA group, and interestingly we observed condensation on the inner surface of the CIT/PLA group. This may be due to low water vapor permeability. Liu et al., (2019) packed *Agaricus bisporus* with gallic acid grafted chitosan film also observed a similar phenomenon. Mushrooms packed in polyethylene (PE) film exhibited the lowest weight loss, again with condensation observed on the surface. Although the MSN/CIT/PLA groups exhibited higher weight loss than the CIT/PLA group, they were still below the market value range (10–12 %) (Liu et al., 2019) at 12 day.

#### 3.2.2 Firmness

Aging of edible mushrooms results in a soft spongy texture characterized by the softening of edible tissue. Therefore, firmness is a key factor reflecting the quality of *Chanterelles*. Fig. 1C depicts the impact of various packing on the firmness of *Chanterelles* during storage.

During storage time, the firmness values of *Chanterelles* were ranged from 10.37 N to 15.12 N. Firmness decreased significantly (*p* < 0.05) in all groups as the number of days increased. This may be due to the occurrence of physiological and biochemical reactions and microbial action in *Chanterelles,* the proteins and polysaccharides were degradation, mycelium was contraction and central vesicles was rupture which reduced the firmness. However, we found that the CIT/PLA group showed the fastest decrease in hardness as the number of days increased, while the 4MSN/CIT/PLA group was the slowest, and the 2MSN/CIT/PLA and 6MSN/CIT/PLA were about the same. Many reasons contribute to the changes in their hardness. Possibly due to the oil content of the essential oils in the film, other researchers suggested that essential oils can slow down the softening process of mushrooms, which may be related to the antibacterial effect of essential oils (Gao et al., 2014, Zou et al., 2023). And Liu et al., (2019) reported that packaging films with low water vapor permeability resulted increased the firmness of *Agaricus bisporus*.

#### 3.2.3 The browning index of Chanterelles

**Fig. 2:**
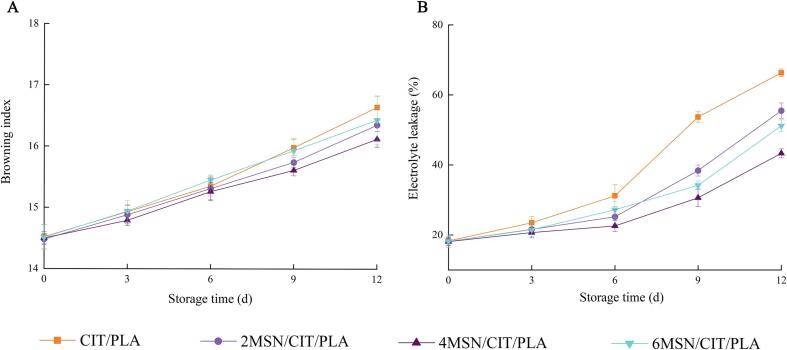
The browning index (A) and electrolyte leakage (B) of *Chanterelles* during storage.

The color of edible mushrooms is a key factor affecting consumer acceptance, which can be quantified by the browning index. The browning index of *Chanterelles* was shown in Fig. 2A.

The browning index of *Chanterelles* were ranged from 14.48 to 16.64, increased in all groups during the storage. It is clear that the MSN/CIT/PLA group had a greater inhibitory effect on the browning of *Chanterelles* than the control group (*p* < 0.05), which could also be observed from the physical appearance of *Chanterelles* (Fig. 1A).

The browning index of MSN/CIT/PLA groups were lower than CIT/PLA group probably because the slow release of citral loaded MSN which kept the citral at a higher level throughout the storage period and had better antioxidant and antibacterial properties, thus inhibit the enzymatic browning reaction and microbial activity of phenolic compounds and delaying the browning of *Chanterelles*. Notably, after 12 days of storage, the browning value of *Chanterelles* treated with 4MSN/CIT/PLA composite film was reduced by about 1.62 times compared to that of *Chanterelles* treated with CIT/PLA composite film, and the browning of *Chanterelles* treated with 4MSN/CIT/PLA composite film was minimal. Oms-Oliu et al., (2010) found that a large leakage of electrolytes caused the browning of mushrooms. In addition, enzymatic browning is due to oxidation catalyzed by polyphenol oxidase (PPO), leading to the oxidation of phenolic compounds to form melanin (Weijn et al., 2013). This will be further confirmed in the next analysis of PPO and POD activities.

#### 3.2.4 Electrolyte leakage

When the protoplasmic membrane structure is disrupted, the permeability of the cell increases and a large amount of intracellular electrolytes are leaked out, resulting in an increase in the electrical conductivity of the cell (Shao et al., 2021). Therefore, conductivity can be used to reflect the integrity of the cell membrane. The relative leakage rates of *Chanterelles* during storage are shown in Fig. 2B.

The increase in electrolyte leakage during storage indicated the loss of cell membrane integrity and reflected the degree of damage to *Chanterelles*. The electrolyte leakage of all treatment groups increased with increasing storage time, which indicated the reduction of cell membrane integrity of *Chanterelles*. After 9 days of storage, the electrolyte leakage of 4MSN/CIT/PLA treated *Chanterelles* (30.6 %) was significantly (*p* < 0.05) lower compared to the control group (53.7 %). At the end of storage, the electrolyte leakage of CIT/PLA treated *Chanterelles* increased to 66.3 %, while that of 4MSN/CIT/PLA treated *Chanterelles* was only 43.3 %, and that of 2MSN/CIT/PLA and 6MSN/CIT/PLA treated *Chanterelles* was 55.5 % and 51.2 %, respectively. The above results indicated that 2MSN/CIT/PLA, 4MSN/CIT/PLA and 6MSN/CIT/PLA treatments could reduce the damage of *Chanterelles* cell membrane, especially the 4MSN/CIT/PLA play an important role in delaying the aging and browning of *Chanterelles*, which might be related to the antioxidant ability of citral and the loading citral to mesoporous silica. Shao et al., (2021) showed that compared to untreated edible mushrooms, edible mushrooms treated with microcapsules of cinnamon essential oil wrapped in starch film had the lowest electrolyte permeability values, the lightest senescence, and the least damaged tissue membrane system of edible mushrooms, and that microcapsules of cinnamon essential oil could reduce cell membrane damage and play an important role in delaying aging and browning of edible mushrooms.

#### 3.2.5 PPO and POD

It has been reported that browning may be caused by the enzymatically catalyzed oxidation of phenolic compounds to produce quinones (Gao et al., 2014). Polyphenol oxidase (PPO) promotes enzymatic browning by catalyzing the oxidation of mono- and di-phenolic substances to produce o-quinones, which in turn leads to browning deterioration, and peroxidase (POD) is closely related to the enzymatic browning and aging process (Gao et al., 2014).

**Fig. 3:**
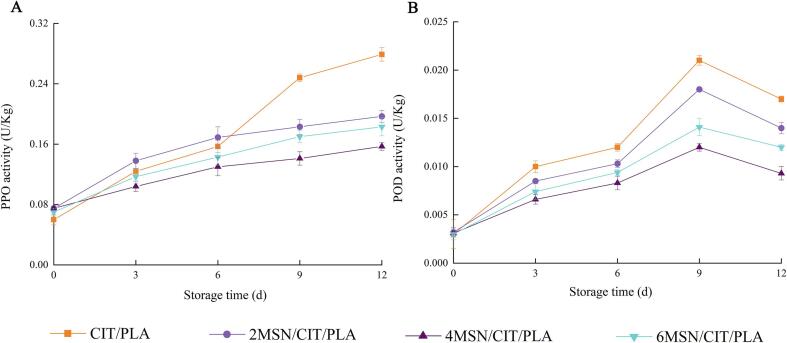
The changes in PPO (A) and POD (B) activity of *Chanterelles* during storage.

Fig. 3A showed the changes of PPO activity of *Chanterelles* during storage. The PPO values of *Chanterelles* increased during storage in all treatment groups. During the first 3 days of storage, PPO increase in CIT/PLA group was not as fast as that in 2MSN/CIT/PLA group, which may be due to the higher release of citral in CIT/PLA group during the first 3 days. After 12 day of storage, the PPO values of 2MSN/CIT/PLA group, 4MSN/CIT/PLA group and 6MSN/CIT/PLA group were significantly lower than that of the CIT/PLA group (*p* < 0.05), which may be related to the fact that the citral level in 2MSN/CIT/PLA group, 4MSN/CIT/PLA group and 6MSN/CIT/PLA group was still high, and citral showed a strong ability to inhibit PPO. Karimirad et al., (2019) found that films made of cumin oil loaded into chitosan nanoparticles were effective in maintaining the levels of antioxidant enzymes in bicarbonate mushrooms.

Fig. 3B showed the changes of POD of *Chanterelles* during storage. The changes in POD in all treatment groups showed an increasing trend followed by a decreasing trend. In the first 3 days of storage, the increasing trend of POD activity of *Chanterelles* treated with CIT/PLA was slower than that of *Chanterelles* treated with 2MSN/CIT/PLA, 4MSN/CIT/PLA and 6MSN/CIT/PLA, which might be due to the greater release of citral in CIT/PLA and stronger antioxidant capacity. However, at the end of storage, the POD activity values of *Chanterelles* treated with 2MSN/CIT/PLA, 4MSN/CIT/PLA and 6MSN/CIT/PLA were significantly (*p* < 0.05) lower than those of *Chanterelles* treated with CIT/PLA, which may be due to the slow release of citral from mesoporous silica. In addition, at day 12, the POD activity values of CIT/PLA group were 1.83 times higher than those of 4MSN/CIT/PLA group (*p* < 0.05); the POD activity values of 2MSN/CIT/PLA group were 1.51 times higher than those of 4MSN/CIT/PLA group (*p* < 0.05); and the POD activity values of 6MSN/CIT/PLA group were 1.29 times higher than those of 4MSN/CIT/PLA group (*p* < 0.05). The increase in POD activity may be due to the oxidation of polyphenols, which leads to the loss of nutritional value, flavor, discoloration, and rapid browning of *Chanterelles*. In conclusion, the inhibitory effect of the 4MSN/CIT/PLA on the PPO and POD may be mainly attributed to the antioxidant capacity of citral and the slow release of citral by mesoporous silica, which enables maintain a high concentration of citral throughout the storage period and can protect the phenolic compounds in *Chanterelles* from being oxidized.

#### 3.2.6 Alpha diversity analysis of bacterial flora

Alpha diversity indices include Chao/Ace and Shannon/Simpson, reflecting species richness and diversity, respectively (Yu et al., 2022). As shown in Table 2, where M is the 4MSN/CIT/PLA group and C is the CIT/PLA group. M0, M3, M6, M9, and M12; C0, C3, C6, C9, and C12 represent *Chanterelles* stored in two films for 0, 3, 6, 9, and 12 days, respectively. The coverage index of each sample ranged from 0.99, which indicated that almost all sequences in the samples were detected.

**Table 2:** Statistical result of bacterial diversity index.

Sample	Shannon	Simpson	Chao1	Ace	Coverage Index
M0	1.51	0.22	834	872	0.99
M3	1.36	0.19	956	1001	0.99
M6	1.67	0.24	921	956	0.99
M9	1.77	0.27	700	737	0.99
M12	1.79	0.26	874	909	0.99
C0	1.05	0.15	860	899	0.99
C3	1.49	0.22	725	758	0.99
C6	1.49	0.21	927	970	0.99
C9	1.59	0.23	809	850	0.99
C12	1.77	0.26	814	849	0.99

Note: M is *Chanterelles* packed with 4MSN/CIT/PLA composite film, C is *Chanterelles* packed with 4CIT/PLA composite film. M0, M3, M6, M9, M12; C0, C3, C6, C9, C12 represent the day 0, 3, 6, 9, and 12 day of *Chanterelles* storage respectively.

For the *Chanterelles* packaged with 4MSN/CIT/PLA composite film, the highest values of Chao1 and Ace indices were found on day 3, which indicated that the surface microbial growth and bacterial abundance increased. However, Chao1 and Ace indices gradually decreased with the extension of storage time. This may be due to the inhibitory effect of citral for the *Chanterelles* packed with CIT/PLA composite film, Chao1 and Ace index also increased and then decreased in the storage period and reached the maximum value on day 6, which may be related to the large release of citral in the early stage. At the later stage of storage, the Chao1 and Ace indices of *Chanterelles* packed with 4MSN/CIT/PLA composite film were lower than those of *Chanterelles* packed with CIT/PLA composite film, indicating that citral was gradually released from mesoporous silica during storage. The slow release of citral played an important role in inhibiting the growth of microorganisms and improving the quality of *Chanterelles*.

With the extension of storage time, the Shannon index of *Chanterelles* packed with CIT/PLA composite film had been showing an increasing trend, indicating that the bacterial diversity on the surface of *Chanterelles* had increased. in contrast, the Shannon index of *Chanterelles* packed with 4MSN/CIT/PLA composite film showed a decreasing and then increasing trend, indicating that the bacterial diversity on the surface of *Chanterelles* had first decreased and then increased. This may be related to the amount of citral released. Simpson index has similar results.

#### 3.2.7 Beta diversity analysis of bacterial flora

**Fig. 4:**
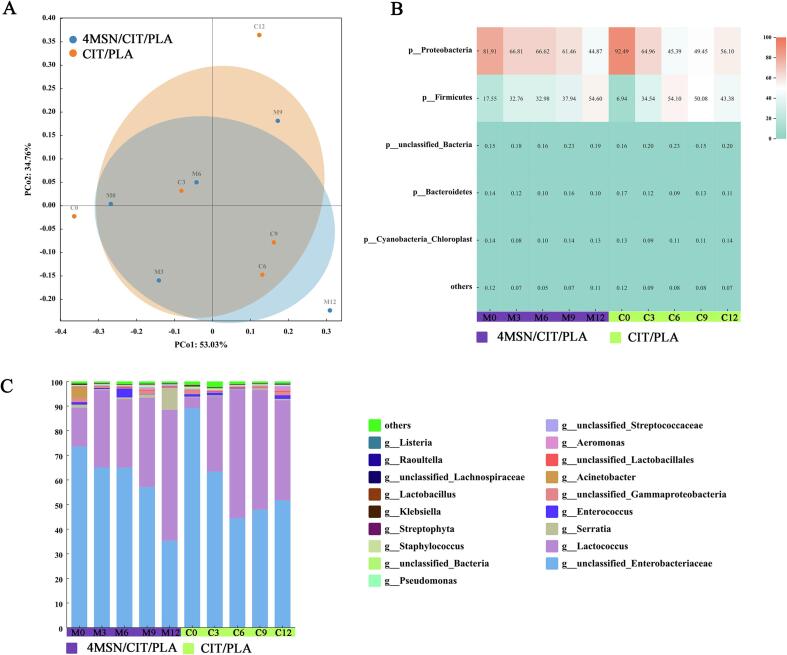
Beta diversity analysis of bacterial communities (A), heat map of relative abundance changes of bacterial communities at the phylum level (B), and community relative abundance composition at the genus level (C) during storage of *Chanterelles*. Bacterial species with relative abundance >0.1 % are labeled in the figure, and ‘other’ indicates relative abundance <0.1 %.

Beta diversity provides a more visual representation of differences in microbial community structure between samples or between sample groups. OTU-based Beta diversity analysis was performed with a distance matrix obtained by the Bray-Curtis statistical calculation method (Fig. 4A).

As can be seen in Fig. 4A, the greater the distance between the samples, the greater the difference in the bacterial community structure, and therefore, the bacterial community structure of *Chanterelles* varied more with the number of days of storage. Especially between 0 day and 12 day, the bacterial colony structure changed greatly in both 4MSN/CIT/PLA and CIT/PLA films, which also indicated that *Chanterelles* was seriously infected by other bacteria during the storage process. We can also see that there is also a significant difference in bacterial flora structure between the samples packed in different films, and the variability is relatively smaller in the 4MSN/CIT/PLA group, especially from 9 day to 12 day when the two groups started to show significant differences, indicating that the 4MSN/CIT/PLA film has better antibacterial effect in the longer storage period.

#### 3.2.8 Changes in relative abundance of bacterial flora

As can be seen from Fig. 4B, that *Proteobacteria* and *Firmicutes* were the most abundant phyla in the *Chanterelles*. And the total percentage of both was above 95 % in each sample. Proteobacteria are related to a wide range of foodborne pathogens. Ban et al., (2022) found a high percentage of *Proteobacteria* in button mushrooms and other fresh produce. At the initial stage, the relative abundance of *Proteobacteria* was the highest in each group of samples*.* However, the relative abundance of *Proteobacteria* showed a decreasing trend, and then *Firmicutes* accounted for an increasing proportion, exceeding *Proteobacteria* at 12 day in the 4MSN/CIT/PLA group, while the CIT/PLA group accounted for the largest proportion at 6 and 9 day. This may be due to the fact that citral is released into *Chanterelles* during storage and is resistant to foodborne pathogens. In particular, the antimicrobial effect of 4MSN/CIT/PLA film was more pronounced at the end of storage. Some studies have shown that essential oils have a variety of antimicrobial mechanisms, such as disrupting enzyme systems or attacking cell membrane structures, destroying lipids and proteins, and disrupting the genetic material of bacteria (Nasiri et al., 2017).

Fig. 4C showed a stacked plot of the relative abundance of *Chanterelles* at the genus level during storage. It can be seen from the Figure that both groups are *unclassified_Enterobacteriaceae* and *Lactococcus* as the dominant role at the genus level throughout storage. In particular, *unclassified_Enterobacteriaceae* accounted for more than 70 % of the total in both groups during the early stages of storage. *Enterobacteriaceae* spp is ubiquitous in nature and is a typical soil bacterium (Su et al., 2021). And *Enterobacteriaceae spp* has a growth-promoting effect on plants*.* Anzuay et al., (2023) found that *Enterobacter* sp. *J49* had a significant yield promoting effect on drought-stressed maize and peanut plants when it was applied to them. Shahid et al., (2019) inoculated *Enterobacter* sp. strain Fs-11 in experimental fields planted with sunflower and found that it could supplement the chemical fertilizer requirement of sunflower and is a potential biofertilizer. Xu et al., (2023) soaked wheat seeds in a solution of *Enterobacter* sp. FN0603 and silicon and then sowed them in pots containing artificial saline soil and found that strain FN0603 and silicon acted in concert to alleviate plant salinity stress and shape the root endophytic community. This may be the environment in which *Chanterelles* grows or there could be *Enterobacteriaceae spp* having a symbiotic relationship with *Chanterelles* to assist the growth. For the 4MSN/CIT/PLA group *Enterobacteriaceae spp* its content decreased rapidly with increasing days to 35.34 % at 12 day, interestingly the lowest was 44.39 % at 3 day for the CIT/PLA group, after which its content increased slightly. This may be due to the fact that *Enterobacteriaceae spp* is also strongly associated with spoilage and coercion of human health. *Enterobacteriaceae spp* were major bacteria in the spoilage of caprine and ovine cheese (Griffin et al., 2020), It is also the main microorganism responsible for the spoilage of dandelion (Dermesonluoglu et al., 2016). *Enterobacteriaceae spp* and so on were the main genera on the surface of the kitchens of food service facilities, posing a certain risk to food safety (Lim et al., 2021), and is also an important cause of healthcare-associated bloodstream infections (BSIs) in children (Boguniewicz et al., 2021). Therefore, it may be that citral had an inhibitory effect on the pathogenic *Enterobacteriaceae spp*, whereas in CIT/PLA films, the citral content decreased after 6 day, leading to an increase in *Enterobacteriaceae spp*. In contrast, the 4MSN/CIT/PLA film had a high level of citral throughout storage due to its slow-release effect.

*Lactococcus* have been shown to be associated with mushroom spoilage, which may lead to mushroom waterlogging and depression lesions (Schill et al., 2021). At the beginning of storage, *Lactococcus* increased in both groups of samples, especially CIT/PLA at a faster rate in the first 6 days, and then slowly decreased. Which may be due to the increase of the dominant spoilage bacteria of *Enterobacteriaceae spp*, which led to its decrease. And for 4MSN/CIT/PLA, its content was increasing, probably due to the more pronounced inhibitory effect of citral on *Enterobacteriaceae spp*. And for *Serratia spp* and *Aeromonas spp* are associated with spoilage of vegetables and considered to be an important foodborne pathogen. There have been found in milk, meat, lettuce, and sausage and is able to grow at 0 °C, making it a potential cause of spoilage of refrigerated foods and possibly causing gastroenteritis in humans (Kim et al., 2022). They were at low levels in both groups of samples and were not the main spoilage bacteria.

## 4 Conclusions

The use of mesoporous silica nanoparticles as a carrier for citral seems to be beneficial for the preservation of mushrooms (*Chanterelles*). The browning index was suppressed, PPO and POD activities were increased, and the rise in electrolyte permeability was retarded in *Chanterelles* packaged in 4MSN/CIT/PLA films, which allowed the *Chanterelles* to maintain better physicochemical properties. Moreover, the main spoilage bacteria (*Enterobacteriaceae spp*) were inhibited and the bacterial community structure was more stable. These findings imply that MSN/CIT/PLA composite films may be used for effective packaging of *Chanterelles* during storage and transportation.

## CRediT authorship contribution statement

**Kai Jiang:** Data curation, Methodology, Investigation, Writing – review & editing. **Bifen Zhu:** Software, Writing – original draft. **Yudi Liu:** Data curation, Investigation, Software. **Haiyan Chen:** Conceptualization, Resources. **Mingwei Yuan:** Resources. **Yuyue Qin:** Conceptualization, Funding acquisition, Project administration, Resources, Supervision, Validation, Writing – review & editing. **Margaret Brennan:** Writing – review & editing, Resources, Supervision. **Charles Brennan:** Writing – review & editing, Resources, Supervision.

## Declaration of Competing Interest

The authors declare that they have no known competing financial interests or personal relationships that could have appeared to influence the work reported in this paper.

## Data Availability

The data that has been used is confidential.

## References

[b0005] Anzuay M.S., Prenollio A., Ludueña L.M., Morla F.D., Cerliani C., Lucero C., Taurian T. (2023). Enterobacter sp. J49: A native plant growth-promoting bacteria as alternative to the application of chemical fertilizers on peanut and maize crops. Current Microbiology.

[b0010] Astm D639-22 (2022).

[b0015] Ban G.-H., Kim B.-K., Kim S.-R., Rhee M.S., Kim S.A. (2022). Bacterial microbiota profiling of oyster mushrooms (Pleurotus ostreatus) based on cultivation methods and distribution channels using high-throughput sequencing. International Journal of Food Microbiology.

[b0020] Boguniewicz J., Revell P.A., Scheurer M.E., Hulten K.G., Palazzi D.L. (2021). Risk factors for microbiologic failure in children with Enterobacter species bacteremia. PLOS ONE.

[b0025] Cui R., Yan J., Cao J., Qin Y., Yuan M., Li L. (2021). Release properties of cinnamaldehyde loaded by montmorillonite in chitosan-based antibacterial food packaging. International Journal of Food Science & Technology.

[b0030] Dermesonluoglu E., Fileri K., Orfanoudaki A., Tsevdou M., Tsironi T., Taoukis P. (2016). Modelling the microbial spoilage and quality decay of pre-packed dandelion leaves as a function of temperature. Journal of Food Engineering.

[b0035] Dong Z., Xu F., Ahmed I., Li Z., Lin H. (2018). Characterization and preservation performance of active polyethylene films containing rosemary and cinnamon essential oils for Pacific white shrimp packaging. Food Control.

[b0040] Gao M., Feng L., Jiang T. (2014). Browning inhibition and quality preservation of button mushroom (Agaricus bisporus) by essential oils fumigation treatment. Food Chemistry.

[b0045] Gong C., Gao W., Wu S. (2023). Inhibitive effects of phytic acid combined with glutathione on the browning and oxidation of King Oyster mushroom (Pleurotus eryngii) slices during drying and storage. Food Chemistry: X.

[b0050] Griffin S., Falzon O., Camilleri K., Valdramidis V.P. (2020). Bacterial and fungal contaminants in caprine and ovine cheese: A meta-analysis assessment. Food Research International.

[b0055] Guzman-Puyol S., Hierrezuelo J., Benítez J.J., Tedeschi G., Porras-Vázquez J.M., Heredia A., Heredia-Guerrero J.A. (2022). Transparent, UV-blocking, and high barrier cellulose-based bioplastics with naringin as active food packaging materials. International Journal of Biological Macromolecules.

[b0060] Jiang K., Li L., Yang Z., Chen H., Qin Y., Brennan C. (2023). Variable characteristics of microbial communities and volatile organic compounds during post-harvest storage of wild morel mushrooms. Postharvest Biology and Technology.

[b0065] Karimirad R., Behnamian M., Dezhsetan S. (2019). Application of chitosan nanoparticles containing Cuminum cyminum oil as a delivery system for shelf life extension of Agaricus bisporus. LWT.

[b0070] Kim J.Y., Jeon E.B., Song M.G., Park S.H., Park S.Y. (2022). Development of predictive growth models of Aeromonas hydrophila on raw tuna Thunnus orientalis as a function of storage temperatures. LWT.

[b0075] Lim E.S., Kim J.J., Sul W.J., Kim J.-S., Kim B., Kim H., Koo O.K. (2021). Metagenomic analysis of microbial composition revealed cross-contamination pathway of bacteria at a foodservice facility. Frontiers in Microbiology.

[b0080] Liu J., Liu S., Zhang X., Kan J., Jin C. (2019). Effect of gallic acid grafted chitosan film packaging on the postharvest quality of white button mushroom (Agaricus bisporus). Postharvest Biology and Technology.

[b0085] Liu Y., Liu R., Shi J., Zhang R., Tang H., Xie C., Jiang L. (2023). Chitosan/esterified chitin nanofibers nanocomposite films incorporated with rose essential oil: Structure, physicochemical characterization, antioxidant and antibacterial properties. Food Chemistry: X.

[b0090] Lu L., Su Y., Xu J., Ning H., Cheng X., Lu L. (2022). Development of gas phase controlled-release antimicrobial and antioxidant packaging film containing carvacrol loaded with HNT-4M(halloysite nanotubes etched by 4 mol/L hydrochloric acid). Food Packaging and Shelf Life.

[b0095] Lu W., Cui R., Zhu B., Qin Y., Cheng G., Li L., Yuan M. (2021). Influence of clove essential oil immobilized in mesoporous silica nanoparticles on the functional properties of poly(lactic acid) biocomposite food packaging film. Journal of Materials Research and Technology.

[b0100] Nasiri M., Barzegar M., Sahari M.A., Niakousari M. (2017). Tragacanth gum containing Zataria multiflora Boiss. Essential oil as a natural preservative for storage of button mushrooms (Agaricus bisporus). Food Hydrocolloids.

[b0105] Nowakowski P., Markiewicz-Żukowska R., Gromkowska-Kępka K., Naliwajko S.K., Moskwa J., Bielecka J., Socha K. (2021). Mushrooms as potential therapeutic agents in the treatment of cancer: Evaluation of anti-glioma effects of Coprinus comatus, Cantharellus cibarius, Lycoperdon perlatum and Lactarius deliciosus extracts. Biomedicine & Pharmacotherapy.

[b0110] Oms-Oliu G., Aguiló-Aguayo I., Martín-Belloso O., Soliva-Fortuny R. (2010). Effects of pulsed light treatments on quality and antioxidant properties of fresh-cut mushrooms (Agaricus bisporus). Postharvest Biology and Technology.

[b0115] Qin Y., Yang J., Xue J. (2015). Characterization of antimicrobial poly(lactic acid)/poly(trimethylene carbonate) films with cinnamaldehyde. Journal of Materials Science.

[b0120] Sattary M., Amini J., Hallaj R. (2020). Antifungal activity of the lemongrass and clove oil encapsulated in mesoporous silica nanoparticles against wheat’s take-all disease. Pesticide Biochemistry and Physiology.

[b0125] Schill S., Stessl B., Meier N., Tichy A., Wagner M., Ludewig M. (2021). Microbiological safety and sensory quality of cultivated mushrooms (Pleurotus eryngii, Pleurotus ostreatus and Lentinula edodes) at retail level and post-retail storage. Foods.

[b0130] Shahid M., Hameed S., Zafar M., Tahir M., Ijaz M., Tariq M., Ali A. (2019). Enterobacter sp. Strain Fs-11 adapted to diverse ecological conditions and promoted sunflower achene yield, nutrient uptake, and oil contents. Brazilian Journal of Microbiology.

[b0135] Shao P., Yu J., Chen H., Gao H. (2021). Development of microcapsule bioactive paper loaded with cinnamon essential oil to improve the quality of edible fungi. Food Packaging and Shelf Life.

[b0140] Sharma P., Ahuja A., Dilsad Izrayeel A.M., Samyn P., Rastogi V.K. (2022). Physicochemical and thermal characterization of poly (3-hydroxybutyrate-co-4-hydroxybutyrate) films incorporating thyme essential oil for active packaging of white bread. Food Control.

[b0145] Si W., Gao Y., Mei X., Wu C., Li J., Zhang J. (2021). Mesoporous silica nanoparticles loaded with capsaicin and their oxidation resistance in meat preservation. Food Chemistry.

[b0150] Su M., Han F., Wang M., Ma J., Wang X., Wang Z., Li Z. (2021). Clay-assisted protection of Enterobacter sp. From Pb (II) stress. Ecotoxicology and Environmental Safety.

[b0155] Tao R., Zhang N., Zhang L., Habumugisha T., Chen Y., Lu Y., Jiang J. (2023). Characterization and antivibrio activity of chitosan-citral Schiff base calcium complex for a calcium citrate sustained release antibacterial agent. International Journal of Biological Macromolecules.

[b0160] Weijn A., Bastiaan-Net S., Wichers H.J., Mes J.J. (2013). Melanin biosynthesis pathway in Agaricus bisporus mushrooms. Fungal Genetics and Biology.

[b0165] Xu D., Gu S., Zhou F., Hu W., Feng K., Chen C., Jiang A. (2021). Mechanism underlying sodium isoascorbate inhibition of browning of fresh-cut mushroom (Agaricus bisporus). Postharvest Biology and Technology.

[b0170] Xu F., Liang Y., Wang X., Guo Y., Tang K., Feng F. (2023). Synergic mitigation of saline-alkaline stress in wheat plant by silicon and Enterobacter sp. FN0603. Frontiers in Microbiology.

[b0175] Yan X., Cheng M., Wang Y., Zhao P., Wang K., Wang Y., Wang J. (2023). Evaluation of film packaging containing mesoporous nanosilica and oregano essential oil for postharvest preservation of mushrooms (Agaricus bisporus). Postharvest Biology and Technology.

[b0180] Yu D., Zhao W., Dong J., Zang J., Regenstein J.M., Jiang Q., Xia W. (2022). Multifunctional bioactive coatings based on water-soluble chitosan with pomegranate peel extract for fish flesh preservation. Food Chemistry.

[b0185] Zawadzka A., Kobus-Cisowska J., Szwajgier D., Szczepaniak O., Szulc P., Siwulski M. (2022). Dual functional cholinesterase inhibitors and complexing of aluminum ions of five species of fungi family depended of drying conditions and extraction process—In vitro study. LWT.

[b0190] Zhang L., Liu Z., Wang X., Dong S., Sun Y., Zhao Z. (2019). The properties of chitosan/zein blend film and effect of film on quality of mushroom (Agaricus bisporus). Postharvest Biology and Technology.

[b0195] Zhu L., Hu W., Murtaza A., Iqbal A., Li J., Zhang J., Pan S. (2022). Eugenol treatment delays the flesh browning of fresh-cut water chestnut (Eleocharis tuberosa) through regulating the metabolisms of phenolics and reactive oxygen species. Food Chemistry: X.

[b0200] Zou Y., Yu Y., Cheng L., Li L., Peng S., Zhou W., Li J. (2023). Effect of citric acid/ pomelo essential oil nanoemulsion combined with high hydrostatic pressure on the quality of banana puree. Food Chemistry: X.

